# Application of artificial intelligence ensemble learning model in early prediction of atrial fibrillation

**DOI:** 10.1186/s12859-021-04000-2

**Published:** 2021-11-08

**Authors:** Cai Wu, Maxwell Hwang, Tian-Hsiang Huang, Yen-Ming J. Chen, Yiu-Jen Chang, Tsung-Han Ho, Jian Huang, Kao-Shing Hwang, Wen-Hsien Ho

**Affiliations:** 1 Department of Hematology, The Fourth Affiliated Hospital of Zhejiang University School of Medicine, No. 1, Shangcheng Road, Yiwu, Zhejiang China; 2 Department of Colorectal Surgery, The Second Affiliated Hospital of Zhejiang University School of Medicine, No. 88, Jiefang Road, Hangzhou, Zhejiang China; 3 Center for Big Data Research, Kaohsiung Medical University, No.100, Shin-Chuan 1st Road, Kaohsiung, 807 Taiwan; 4 Department of Logistics Management, National Kaohsiung University of Science and Technology, No.1, University Road, Kaohsiung, 824 Taiwan; 5 Department of Healthcare Administration and Medical Informatics, Kaohsiung Medical University, No.100, Shin-Chuan 1st Road, Kaohsiung, 807 Taiwan; 6 Department of Engineering Science, National Cheng Kung University, No.1, University Road, Tainan, 701 Taiwan; 7 Department of Electrical Engineering, National Sun Yat-Sen University, No.70, Lienhai Road, Kaohsiung, 804 Taiwan; 8 Department of Medical Research, Kaohsiung Medical University Hospital, No.100, Shin-Chuan 1st Road, Kaohsiung, 807 Taiwan

**Keywords:** Atrial fibrillation, Electrocardiogram, Artificial intelligence, Ensemble learning

## Abstract

**Background:**

Atrial fibrillation is a paroxysmal heart disease without any obvious symptoms for most people during the onset. The electrocardiogram (ECG) at the time other than the onset of this disease is not significantly different from that of normal people, which makes it difficult to detect and diagnose. However, if atrial fibrillation is not detected and treated early, it tends to worsen the condition and increase the possibility of stroke. In this paper, P-wave morphology parameters and heart rate variability feature parameters were simultaneously extracted from the ECG. A total of 31 parameters were used as input variables to perform the modeling of artificial intelligence ensemble learning model.

**Results:**

This paper applied three artificial intelligence ensemble learning methods, namely Bagging ensemble learning method, AdaBoost ensemble learning method, and Stacking ensemble learning method. The prediction results of these three artificial intelligence ensemble learning methods were compared. As a result of the comparison, the Stacking ensemble learning method combined with various models finally obtained the best prediction effect with the accuracy of 92%, sensitivity of 88%, specificity of 96%, positive predictive value of 95.7%, negative predictive value of 88.9%, F1 score of 0.9231 and area under receiver operating characteristic curve value of 0.911.

**Conclusion:**

In feature extraction, this paper combined P-wave morphology parameters and heart rate variability parameters as input parameters for model training, and validated the value of the proposed parameters combination for the improvement of the model’s predicting effect. In the calculation of the P-wave morphology parameters, the hybrid Taguchi-genetic algorithm was used to obtain more accurate Gaussian function fitting parameters. The prediction model was trained using the Stacking ensemble learning method, so that the model accuracy had better results, which can further improve the early prediction of atrial fibrillation.

## Background

Atrial fibrillation is a paroxysmal heart disease most commonly found in clinical arrhythmias and is characterized by rapid and irregular beating of the heart. Due to the irregular heart beating, it is easy for irregular blood flow to produce blood clots and increase the possibility of stroke, heart failure and dementia [1]. According to U.S. statistics, 45% of the patients are over 75 years of age, with a total prevalence of 0.95%, and the higher the age, the higher the prevalence, from 0.1% under 55 to 9% over 80. It is estimated the total prevalence will increase 2.4-fold by 2050, and the prevalence of adults over 80 years of age will exceed 50% [2]. In other statistics, atrial fibrillation is also highly correlated with hypertension, diabetes, excessive drinking, and other heart diseases such as valvular heart disease, heart failure, coronary artery disease, and the prevalence rate of Male is higher than that of Female [3].

Atrial fibrillation occurs due to abnormal discharge of the atrial wall tissue, which affects the overall electrophysiological response of the heart, leading to irregular heartbeats. It is divided into three types according to its severity and duration: (1) paroxysmal atrial fibrillation, which lasts no more than 7 days, and usually recovers within 24 h per episode; (2) persistent atrial fibrillation, which lasts more than 7 days, is not easy to recover on its own, and requires medication and electric shock rectification for recovery; (3) permanent atrial fibrillation, which lasts more than 1 year, cannot be restored by drugs and electric shock rectification [1]. Canadian studies have shown that patients with paroxysmal and persistent atrial fibrillation are likely to deteriorate into permanent atrial fibrillation within a few years, and the degree of deterioration is related to age, heart rate and cardiomyopathy [4]. And regardless of the atrial fibrillation at any stage, most patients are asymptomatic during the onset, and only a small number of people experience chest pain, palpitations or dyspnea, so most people do not know that they have atrial fibrillation, resulting that the disease treatment can not start early so it evolves into intractable permanent atrial fibrillation [5].

The current clinical diagnosis of atrial fibrillation is through electrocardiogram (ECG) detection. However, in patients with paroxysmal atrial fibrillation not in the onset, the rhythm of the ECG is not significantly different from that of normal people, leading to difficulties in diagnosing atrial fibrillation [6]. In recent years, artificial intelligence and machine learning have developed vigorously, and have a considerable impact in the medical field, including smart diagnosis, medical image processing and classification, drug development testing and nutrition recommendations, etc. [7]. Especially in terms of smart diagnosis, many scholars hope to use the various data provided by patients, including basic health data, family history, various medical image file cases, and complaints at the clinic, etc., to achieve intelligent diagnosis and treatment through machine learning modeling [8].

In intelligent diagnosis, the establishment of a classification model is also a very important part. After cases and parameters are collected, and the different training algorithms of the model are calculated, it will be able to analyze the input data and calculate the most possible result, i.e. whether atrial fibrillation is present [9].

For model selection, most literatures use a single model for prediction, such as decision tree, support vector machine, k nearest neighbor, artificial neural network, etc. But, in recent years, ensemble learning algorithms have gradually received attention. The training results of a model may be affected by its own algorithm and data set, resulting in poor accuracy or prediction result. Under such a circumstance, if multiple models are jointly trained and decided, theoretically, the overall prediction accuracy can be improved [10, 11].

There are two concepts of ensemble learning methods: (1) decision-making by training multiple models; (2) repeated sampling of the training set to increase the number of models trained. Multiple models in the ensemble learning method can be the same classification model such as Bagging ensemble learning method and AdaBoost ensemble learning method, and they can also be different models, such as Stacking ensemble learning method [10]. There are also considerable applications of ensemble learning in the medical field. For the prediction of atrial fibrillation, Zhang and Zhu [12] used the XGBoost integrated classifier to detect atrial fibrillation by decomposing ECG signals; Firoozabadi et al. [13] applied the decision tree of the bagged trees classifier to P-wave and interbeat interval features to classify whether or not atrial fibrillation is present; the features selected by Zabihi et al. [14] come from the time, frequency, time–frequency domains, and phase space reconstruction of the ECG signals, and then they used a random forest classifier to classify selected features to predict atrial fibrillation.

In terms of parameter selection, it can be roughly divided into two categories: (1) using P-wave waveform feature parameters; (2) using heart rate variability parameters as input parameters of artificial intelligence models. However, under extensive search of existing reference literature, there is no relevant literature that combines these two types of parameters at the same time as input parameters of artificial intelligence models to predict whether they are patients with atrial fibrillation. In research, the parameters are preferably multi-domain, representative and low isomorphism, which will be more helpful for the training of the model, so this paper attempted to combine the two types of parameters to train together for good results of the establishment of artificial intelligence models. Therefore, this paper used various ECG characteristics parameters of atrial fibrillation to perform artificial intelligence model training on data, and used Bagging ensemble learning method, AdaBoost ensemble learning method and Stacking ensemble learning method to compare and improve the model prediction accuracy.

## Methods

### Data set description

The data set used in this paper is an online database provided by Physionet (https://physionet.org/physiobank/database/afpdb/), of which the atrial fibrillation paroxysmal database (AFPDB) is the most commonly used database in atrial fibrillation research. This database contains the ECG data of 50 normal people and 50 patients with paroxysmal atrial fibrillation. Each data report contains a 30-min record with no obvious episode of atrial fibrillation. In this study, the lead II ECG signal was used, and its sampling frequency was 128 Hz [15].

### Feature extractions

The extraction of ECG feature parameters is explained in two parts, which are the P-wave morphology parameters and the heart rate variability parameters. In this paper, a total of 6 P-wave morphology parameters (as shown in Table 1) and 25 heart rate variability parameters (as shown in Table 2) were obtained. The heart rate variability parameters were (1) 11 time-domain parameters, (2) 7 frequency-domain parameters, and (3) 7 non-linear parameters. A total of 31 parameters were used as input values for the atrial fibrillation classification prediction model, as shown in Table 1. The method for extracting ECG feature parameters is described below.

**Table 1:** P-wave morphology feature parameters of ECG

Parameter	Units	Description
PW	[ms]	Width of the P-wave measured for a particular heart pulse
PA	[mV]	Amplitude of the P-wave
PD	[ms]	Time distance of the beginning of the P-wave till its maximum
*A*	–	Parameters of the P-wave fitted Gaussian function
*C*	–	Parameters of the P-wave fitted Gaussian function
*W*	–	Parameters of the P-wave fitted Gaussian function

**Table 2:** Heart rate variability feature parameters of ECG

Parameter	Units	Description
Time-domain		
$\stackrel{-}{\mathrm{RR}}$	[ms]	The mean of RR intervals
SDNN	[ms]	Standard deviation of normal to normal RR intervals
$\stackrel{-}{\mathrm{HR}}$	[1/min]	The mean heart rate
SDHR	[1/min]	Standard deviation of instantaneous heart rate values
MinHR	[beats/min]	Min heart rate per minute
MaxHR	[beats/min]	Maximum heart rate per minute
RMSSD	[ms]	The root mean square of successive RR interval differences
NN50	[count]	Number of successive RR interval pairs that differ more than 50 ms
pNN50	[%]	NN50 divided by the total number of all NN intervals
HRV triangular index	–	The integral of the RR interval histogram divided by the height of the histogram
TINN	[ms]	Baseline width of the NN interval histogram
Frequency-domain		
VLF power	[ms^2^]	Absolute power of VLF band
LF power	[ms^2^]	Absolute power of LF band
HF power	[ms^2^]	Absolute power of HF band
LF/HF	–	Ratio between LF and HF band powers
Total power (TP)	[ms^2^]	Total spectral power
Normalized LFP	–	LF/(TP-VLF)
Normalized HFP	–	HF/(TP-VLF)
Nonlinear		
SD1	[ms]	Poincaré plot standard deviation perpendicular the line of identity
SD2	[ms]	Poincaré plot standard deviation along the line of identity
SD2/SD1	[%]	Ratio of SD2 to SD1
ApEn	–	Approximate entropy
SampEn	–	Sample entropy
${\alpha }_{1}$,${\alpha }_{2}$	–	Short-term and long-term fluctuations of detrended fluctuation analysis (DFA)

### P-wave morphology methods

In this paper, the P-wave morphology captured the P-wave width of the P-wave measured for a particular heart pulse (PW), the amplitude of the P-wave (PA), and the time distance of the beginning of the P-wave till its maximum (PD) [16]. There are also literatures on the method of P-wave fitting for Gaussian function, which obtains the variables of the Gaussian function as morphology parameters [17–20]. The Gaussian function fitted by the P-wave is expressed as $y(i)=A\bullet {e}^{{-\left(\frac{i-C}{W}\right)}^{2}}$, $i=\mathrm{1,2},\dots ,D$, *D* as the total number of all data points of the P wave, where *A, C* and *W* are the parameters of the Gaussian function.

P-wave fitting of Gaussian function is an optimization problem in itself, that is, the fitting error value is minimized, so a better fitting waveform can be obtained through the optimization method. The author of this paper has applied the hybrid Taguchi-genetic algorithm [21–25] to P-wave fitting, to search for the three parameters *A, C* and *W* of the best Gaussian function to obtain an optimized P-wave fitting Gaussian function Curve. Please refer to Tang et al. [26] for its optimized P-wave fitting method.

### Time-domain methods

The time-domain method is simple to calculate and can be directly applied to a series of continuous RR (R-wave to R-wave) intervals [16]. The most obvious measures are the mean hear rate per minute ($\mathrm{HR}$) and the standard deviation of instantaneous heart rate values (SDHR), the maximum heart rate per minute (MaxHR), the minimum heart rate per minute (MinHR) and the mean of RR intervals ($\stackrel{-}{\mathrm{RR}}$). The standard deviation of the normal to normal RR intervals reflects the overall (short-term and long-term) changes (SDNN) within the RR interval series. The root mean square of successive RR interval differences (RMSSD) can be used to measure short-term variability. Another measurement method calculated based on the difference between successive RR intervals is NN50, which is a difference between successive intervals of more than 50 ms or the corresponding relative amount pNN50, which is NN50 divided by the total number of all NN intervals. In addition to the above statistical parameters, two geometric measures are calculated according to the RR interval histogram as (1) the heart rate variability (HRV) triangular index and (2) the baseline width of the RR histogram evaluated through the triangular interpolation of the NN interval histogram (TINN).

### Frequency-domain methods

In the frequency-domain method, a spectrum estimate is calculated for the RR interval sequence [27]. Before spectral estimation, the RR interval sequence is converted to an equidistant sampling sequence by cubic spline interpolation. The frequency spectrum is estimated by two different methods: Welch’s periodogram and autoregressive (AR) modelling. In Welch’s periodogram, the RR series is divided into multiple overlapping segments, each segment is windowed to reduce the leakage effect, and the spectrum estimate is obtained by averaging the fast Fourier transform (FFT) spectrum of these windowed segments. In the AR modelling, an AR model of a specific order is used to model the RR series, and the spectrum estimate is obtained from the estimated model parameters. The AR spectrum can be divided into different spectral components by applying spectral decomposition.

Then the spectrum estimation is divided into very low frequency (VLF), low frequency (LF) and high frequency (HF) bands. In the case of short-term HRV recordings in normal human subjects, the common limits for these bands are 0–0.04 Hz (VLF), 0.04–0.15 Hz (LF), and 0.15–0.4 Hz (HF). Maximum power (including VLF power, LF power, HF power), LF/HF power ratio, and the total spectral power (TP) of the HRV measurement values are extracted from the VLF, LF, and HF bands respectively.

### Nonlinear methods

Among the nonlinear heart rate variability parameters, the most common is Poincaré plot [28]. The distribution chart is made by the length of the distance between two adjacent RRs. The chaos and randomness of the time series can be observed from this chart, where the ellipse distribution has a short-axis radius of SD1 and a long-axis radius of SD2.

Poincaré plots show parasympathetic nerve activity in humans in the clinic. The study of Park et al. [29] also used the two parameters SD1 and SD2 obtained by this algorithm as important indicators for atrial fibrillation detection, indicating that these parameters are helpful for the prediction of atrial fibrillation.

Entropy is an indicator to judge whether the data is regular. The larger the entropy, the more irregular and unpredictable the data set. Approximate entropy (ApEn) is a method to quantify the irregularity and unpredictability of time series data, and a practical method and indicator for analyzing medical data. The calculation method of the sample entropy (SampEn) is similar to that of approximate entropy.

Detrended fluctuation analysis (DFA) can calculate the long-range correlation and short-range autocorrelation of time series. The slopes ${\alpha }_{1}$ and ${\alpha }_{2}$ of the short-range and long-range distribution points after first-order linear fitting by the least square method are used as the correlation indicators [30]. This stage shows the nonlinear parameters common in heart rate variability analysis.

### Ensemble learning modeling method

Each classification model has its advantages and disadvantages, suitable data domains and data volumes. If it can combine multiple classifiers to make joint decisions, prediction accuracy will be improved. This is the concept of ensemble learning. Common learning and common decision-making of multiple models makes the classification model more robust, and combines multiple weak classifiers into a strong classifier, but there are two conditions for ensemble learning to make the classification result better: (1) There must be a difference between each classifier; (2) The accuracy of each classifier should be > 0.5. If both conditions are met, the more classifiers are combined, theoretically the better the prediction accuracy [31].

In this study, four classifiers, decision tree, k-Nearest Neighbor, artificial neural network and support vector machine were evaluated. But ultimately, 100 decision trees of CART algorithm with 0.1 complexity parameter (default values is 0.01) were chosen in following ensemble learning processes.

There are three methods most commonly used in ensemble learning: (1) Bagging ensemble learning method, (2) AdaBoost ensemble learning method, and (3) Stacking ensemble learning method. At first, the implement steps of Bagging ensemble learning method are (1) to generate randomly bootstrapped samples from the given dataset; (2) to train *N* classifiers by samples generated from Step 1; (3) to repeat Steps 1 and 2 until accuracy of every classifier is larger than 50%. Then, the implement steps of AdaBoost ensemble learning method are (1) to set same sampling weight for all samples; (2) to generate a randomly bootstrapped sample from the given dataset; (3) to train a classifier by the bootstrapped sample; (4) to evaluate accuracy of the classifier, if smaller than 50%, then return to Step 2; (5) to reduce the sampling weight of correctly classified samples for this classify model; (6) to normalize the sampling weight of all samples; (7) to repeat Steps 2 to 6 until *N* classifiers are generated.

With regard to Stacking ensemble learning method, we simultaneously train multiple different classifier models by all training data. Then, we use the prediction results of all classifier models as the input value of the next-layer logical regression model. Finally, we use this two-layer stacking method to predict data classification. This paper compared the prediction effects of these three ensemble learning algorithms.

### Evaluation method

There are 7 kinds of model performance indicators used in this paper, which are accuracy, sensitivity, specificity, positive predictive value, negative predictive value, F1 score and area under receiver operating characteristic curve (AUROC).

These indicators are the most commonly used in modeling to evaluate the advantages and disadvantages of models [31]. This paper used these indicators to compare the advantages and disadvantages of the models to find the most suitable model for the classification and prediction for atrial fibrillation.

## Results

**Fig. 1:**
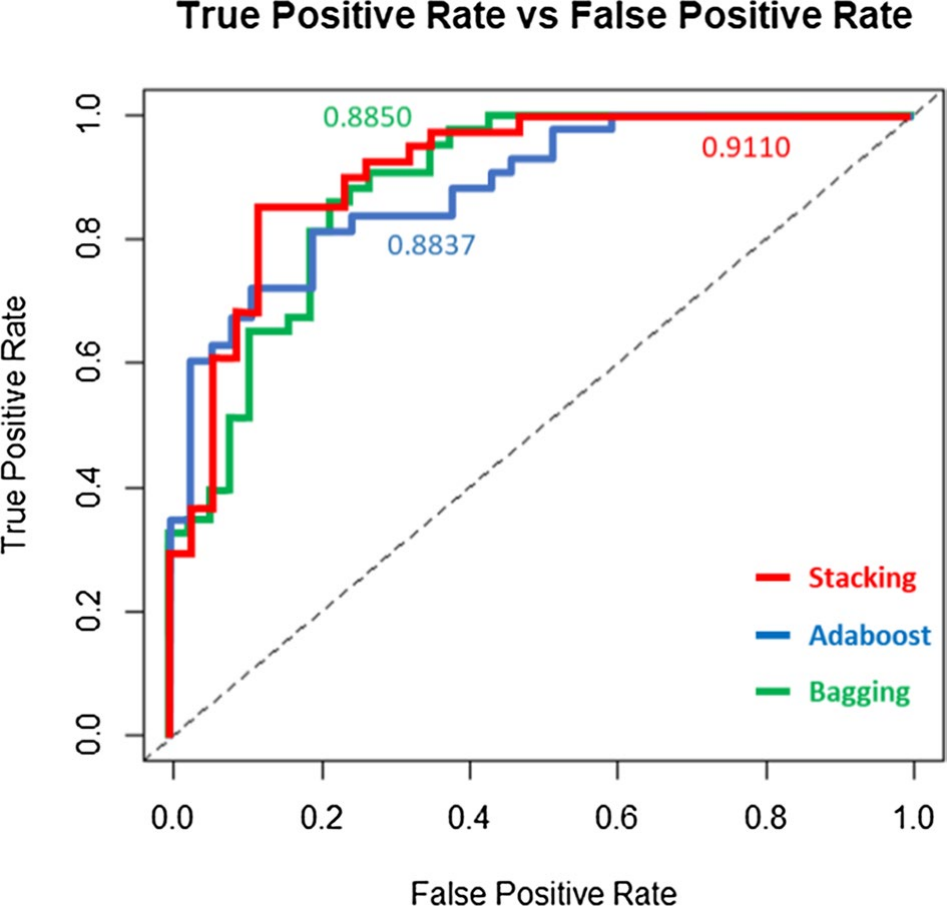
ROC curves of three ensemble learning models

In the study of the method of extracting the eigenvalues of ECG signals, this paper combined P-wave morphology parameters and heart rate variability parameters. According to the principle of artificial intelligence algorithm parameter selection, multi-parameters can provide more diversified basis for model judgement [32, 33], and can effectively improve the accuracy of model judgment. This paper used the technology of ensemble learning on modelling, trained multiple models together, and finally made a joint decision on early prediction of atrial fibrillation. In this paper, three different ensemble learning methods such as Bagging ensemble learning method, AdaBoost ensemble learning method and Stacking ensemble learning method were used, and different classifiers were combined to perform experiments to find the optimal model as the classification model for assisting the early diagnosis of atrial fibrillation patients. Then the accuracies of different prediction models were compared. The models were trained and verified using tenfold cross validation. All models were built using the R programming language. The experimental results are shown in Table 3, where AUROC can be calculated from the area under the ROC curve in Fig. 1.

**Table 3:** Comparison of prediction results of three ensemble learning models

Ensemble learning model	Accuracy	Sensitivity	Specificity	Positive predictive value	Negative predictive value	F1 score	AUROC
Bagging	0.89	0.82	0.96	0.9535	0.8421	0.8972	0.8850
AdaBoost	0.88	0.82	0.94	0.9318	0.8393	0.8868	0.8837
Stacking	0.92	0.88	0.96	0.9565	0.8889	0.9231	0.9110

## Discussion

From Table 3, it can be found that all the models built using ensemble learning had good results. The method with the highest accuracy rate was Stacking ensemble learning method, which had a classification accuracy of 92%, a sensitivity of 88%, and a positive prediction value of 96%. As the purpose of this paper is to hope that all people with atrial fibrillation should be screened, high sensitivity should be required. This paper can successfully identify up to 88% of patients with atrial fibrillation, while the accuracy for judging healthy people was also as high as 96%.

In this paper, the reason why the AdaBoost ensemble learning method was not as effective as the Bagging ensemble learning method may be that the training samples contained outliers and unreliable samples, which led to the model training tending to adapt to unreliable samples, so AdaBoost ensemble learning method cannot effectively improve the accuracy compared to the Bagging ensemble learning method [10]. Finally, the Stacking ensemble learning method was used to combine the Bagging ensemble learning method and the AdaBoost ensemble learning method, and the highest accuracy of 92% was obtained.

## Conclusions

In this paper, an artificial intelligence model with high accuracy was completed with the signals of ECG through feature extraction and ensemble learning model planning. In feature extraction, this paper combined P-wave morphology parameters and heart rate variability parameters as input parameters for model training, and validated the value of the proposed parameters combination for the improvement of the model’s predicting effect. In the calculation of the P-wave morphology parameters, the hybrid Taguchi-genetic algorithm was used to obtain more accurate Gaussian function fitting parameters. The prediction model was trained using the Stacking ensemble learning method, which made the model accuracy better with the accuracy of 92%, sensitivity of 88%, specificity of 96%, positive predictive value of 95.7%, negative predictive value of 88.9%, F1 score of 0.9231 and AUROC value of 0.911. Because it is very difficult to detect atrial fibrillation while the disease is not onset, it is almost impossible for any doctor to learn from the ECG signal with naked eyes. The artificial intelligence model established in this paper can be an important tool in the early screening of atrial fibrillation, and provide a reference for diagnosis by the doctors for early interventional treatment to avoid deterioration of the condition.

## Data Availability

The datasets analysed during the current study are available in the atrial fibrillation paroxysmal database (AFPDB) repository, https://physionet.org/physiobank/database/challenge/2016/.

## Abbreviations

AFPDB: Atrial fibrillation paroxysmal database
ApEn: Approximate entropy
AR: Autoregressive
AUROC: Area under receiver operating characteristic curve
DFA: Detrended fluctuation analysis
ECG: Electrocardiogram
FFT: Fast Fourier transform
HF: High frequency
HRV: Heart rate variability
LF: Low frequency
SampEn: Sample entropy
VLF: Very low frequency
